# Connexin 43 and Pannexin 1 hemichannels as endogenous regulators of innate immunity in sepsis

**DOI:** 10.3389/fimmu.2024.1523306

**Published:** 2024-12-23

**Authors:** Jianhua Li, Li Lou, Weiqiang Chen, Xiaoling Qiang, Cassie Zhu, Haichao Wang

**Affiliations:** ^1^ The Feinstein Institutes for Medical Research, Northwell Health, Manhasset, NY, United States; ^2^ Department of Emergency Medicine, Donald and Barbara Zucker School of Medicine at Hofstra/Northwell, Hempstead, NY, United States

**Keywords:** Connexin 43, Pannexin 1, hemichannel, ATP, HMGB1, inflammasome, mimetic peptide, innate immune cells

## Abstract

Sepsis is a life-threatening organ dysfunction resulting from a dysregulated host response to infections that is initiated by the body’s innate immune system. Nearly a decade ago, we discovered that bacterial lipopolysaccharide (LPS) and serum amyloid A (SAA) upregulated Connexin 43 (Cx43) and Pannexin 1 (Panx1) hemichannels in macrophages. When overexpressed, these hemichannels contribute to sepsis pathogenesis by promoting ATP efflux, which intensifies the double-stranded RNA-activated protein kinase R (PKR)-dependent inflammasome activation, pyroptosis, and the release of pathogenic damage-associated molecular pattern (DAMP) molecules, such as HMGB1. Mimetic peptides targeting specific regions of Cx43 and Panx1 can distinctly modulate hemichannel activity *in vitro*, and diversely impact sepsis-induced lethality *in vivo*. Along with extensive supporting evidence from others, we now propose that hemichannel molecules play critical roles as endogenous regulators of innate immunity in sepsis.

## Introduction

Microbial infections and resultant sepsis account for nearly 20% of deaths worldwide (1), and annually cost over $60 billion in healthcare in the U.S. alone. Despite the urgent need, effective therapies remain elusive, as setbacks in sepsis clinical trials have dampened enthusiasm for both preclinical investigation of complex sepsis pathophysiology and clinical development of innovative sepsis therapies. However, prior pre-clinical studies of pathogenic cytokines (e.g., TNF) in inflammatory diseases (2) have led to the Nobel Prize -Winning anti-TNF therapies for patients with rheumatoid arthritis (3, 4), inspiring an improved understanding of the complex pathogenic mechanism of sepsis. As a multifactorial disorder, sepsis is initiated by the innate immune system, the body’s first line of defense against microbial infections (5, 6).

Monocytes, originating from hematopoietic stem cells in the bone marrow, circulate continuously in the bloodstream to search for invading pathogens or damaged tissues. Upon detecting pathogen- or damage-associated molecular pattern molecules (PAMPs or DAMPs), they infiltrate affected tissues and differentiate into resident macrophages (7, 8). Macrophages and monocytes are equipped with various pattern recognition receptors (PRR) such as the Toll-like receptor 4 (TLR4) and TLR9 (9–11) to detect PAMPs, including bacterial lipopolysaccharides (LPS) and CpG-DNA (12, 13). For instance, upon encountering small amounts of LPS, an LPS-binding protein (LBP) (14) aids in its delivery to a co-receptor, cluster of differentiation 14 (CD14) (15), which then presents it to TLR4 (10). This interaction rapidly initiates the release of “early” proinflammatory cytokines such as tumor necrosis factor (TNF) (2), interleukin-1β (IL-1β) (16) and interferon-γ (IFN-γ) (17). While early cytokine responses can be protective against microbial infections (18–21), excessive cytokine production (e.g., TNF) can contribute to the pathogenesis of sepsis (22) and septic shock (2). Despite attempts to neutralize TNF in clinical settings, therapeutic windows for early cytokines are narrow, motivating the search for “late” mediators that may provide broader therapeutic opportunities in treating lethal infections. In 1999, we discovered that high mobility group box 1 (HMGB1) was released by endotoxin-stimulated macrophages and monocytes, and functioned as a late mediator of lethal endotoxemia (23). In animal models of lethal sepsis induced by cecal ligation and puncture (CLP), circulating HMGB1 levels peaked between 24-36 hours (24). Pharmacological inhibition of HMGB1 with neutralizing antibodies conferred protection against lethal sepsis, even when administered 24 hours post-CLP (24, 25), establishing HMGB1 as a “late” mediator of sepsis with a broader therapeutic window (26–30).

## Role of inflammasome activation in the regulation of HMGB1 release

**Figure 1:**
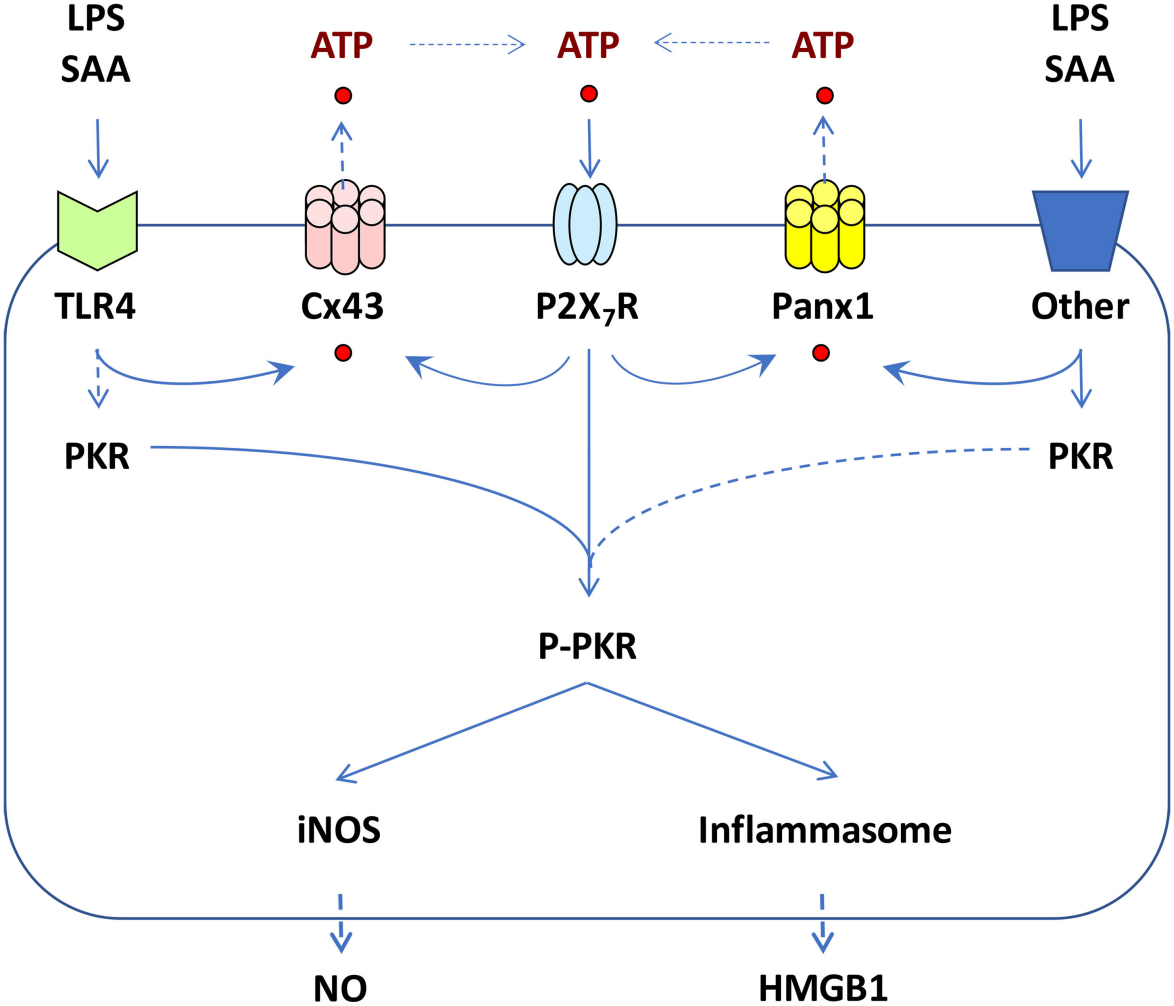
Proposed roles of Cx43 and Panx1 hemichannels in regulating ATP release and inflammasome activation. Prolonged exposure to crude LPS or SAA induced upregulation of Cx43 and Panx1 hemichannels, potentially contributing to extracellular ATP efflux, P2X7R-mediated PKR phosphorylation, pyroptosis, and the release of DAMPs such as HMGB1. Concurrently, PKR activation led to increased expression of inducible nitric oxide synthase (iNOS) and parallel production of nitric oxide (NO) in macrophage cultures.

Lacking a signal sequence, HMGB1 cannot be secreted through the classical Endoplasmic Reticulum (ER) - Golgi exocytotic pathways (23). Instead, inflammatory stimuli promoted HMGB1 acetylation (31), lactylation (31) or phosphorylation (32, 33), facilitating its translocation to cytoplasmic vesicles and secretion through non-classical exosomal secretory pathways (31, 34–37). In addition, HMGB1 can be passively released via pyroptosis (**Figure 1**) (38, 39), a programmed necrotic cell death pathway mediated by Casp-1-dependent canonical and Casp-11/4/5-dependent non-canonical inflammasome activation pathways (40).

Accumulative evidence has supported the critical role of inflammasome activation in regulating LPS/ATP-induced HMGB1 release, as genetic disruption of key inflammasome components like Casp-1 or Nalp3 significantly impairs this process (41). Additionally, the double-stranded RNA-activated protein kinase R (PKR) has been identified as a key regulator of inflammasome activation and HMGB1 release (**Figure 1**) (42). Genetic deletion of PKR expression or pharmacological inhibition of its phosphorylation similarly disrupts inflammasome activation, pyroptosis, and HMGB1 release (42, 43). Notably, bacterial toxins are believed to activate inflammasome signaling pathways partly by triggering passive ATP leakage (44). Indeed, ultrapure LPS, free of contaminating bacterial proteins and nucleic acids, can induce early cytokine production (e.g., TNF), but cannot trigger nitric oxide (NO) production and HMGB1 secretion unless the LPS priming is accompanied by a second stimulus, ATP (41, 42, 45, 46). Similarly, while ATP itself can induce PKR phosphorylation (42), it cannot independently trigger the release of HMGB1 or other inflammasome-dependent cytokines (e.g., IL-1β and IL-18) without prior LPS priming (41, 47–49).

## Role of extracellular ATP in the regulation of inflammasome activation

Extracellular ATP is believed to activate the inflammasome by binding to the purinergic P2X7 receptor (P2X7R) (50), which rapidly opens the ATP-gated P2X7R channel, permitting the influx of small cationic ions. This activation subsequently recruits and activates connexin 43 (Cx43) and pannexin 1 (Panx1) hemichannels (**Figure 1**), allowing the passage of larger anionic molecules up to 900 Da, including ATP (51–53). This ATP-mediated, feed-forward release of ATP amplifies LPS-stimulated inflammasome activation (54) and subsequent release of inflammasome-dependent cytokines (e.g., IL-1β and IL-18) (47–49, 55).

Our previous work demonstrated that both crude LPS and purified SAA upregulated the expression of Cx43 and Panx1 hemichannels in macrophage cultures (56, 57), and effectively induced PKR-dependent inflammasome activation and HMGB1 release (**Figure 1**) (56). Connexins and pannexins are transmembrane proteins that form hemichannels on the surface of monocytes and macrophages. When hemichannels from neighboring cells align, they can create a gap junction (GJ), enabling direct exchange of ions, small molecules, and signaling molecules between the cytoplasm of adjacent cells. However, hemichannels also exist in an unpaired state on the cell surface, typically remaining closed in quiescent cells to prevent uncontrolled release of intracellular contents. In response to inflammatory signals, however, these hemichannels can open, allowing ions and small molecules—such as ATP—to pass between the intracellular and extracellular spaces. Among hemichannel proteins, Cx43 and Panx1 are the most extensively studied, especially in macrophages and monocytes, where they play critical roles in regulating immune activation and cytokine production.

In light of our observation that a hemichannel blocker, carbenoxolone (CBX), dose-dependently inhibited both endotoxin- and SAA-induced PKR activation and HMGB1 release (56, 58, 59), we proposed that hemichannel molecules like Cx43 and Panx1 may play important roles in regulating the release of ATP, which intensifies inflammatory responses through PKR-dependent inflammasome activation, pyroptosis, and HMGB1 release (**Figure 1**). Below we discuss the distinct and shared roles of Cx43 and Panx1 in modulating innate immunity during sepsis, emphasizing their tissue-specific contributions to inflammation and sepsis progression.

## Role of Cx43 as an endogenous regulator of innate immunity in sepsis

Cx43, a member of the connexin family, is widely expressed in innate immune cells such as monocytes (60) and macrophages (61). In sepsis, Cx43 forms unpaired hemichannels that enable the release of signaling molecules, such as ATP, into the extracellular space, thereby amplifying innate immune responses.

### Up-regulation of Cx43

To investigate the potential role of Cx43 hemichannel in regulating innate immunity, we measured Cx43 expression in wild-type and TLR4-deficient macrophages after prolonged stimulation with crude endotoxins or highly purified human serum amyloid A (SAA) (56). While quiescent macrophages constitutively expressed Cx43 at relative low levels, exposure to crude LPS and SAA resulted in a marked increase in Cx43 expression (by 45-60 folds) in wild-type, but not in the TLR4-deficient macrophages, suggesting that LPS and SAA induced Cx43 expression in a TLR4-dependent fashion (**Figure 1**) (56). Our findings were consistent with previous studies showing that bacterial endotoxins and proinflammatory cytokines (e.g., TNF and IFN-γ) up-regulated Cx43 expression in macrophages/monocytes (60, 62) and Kupffer cells (63). Given the expression of Cx43 in Kupffer cells (63)—the liver’s resident macrophages that play a crucial role in mediating hepatic immune responses—it is plausible that Cx43 may also play a significant role in regulating inflammasome activation in these cells. Indeed, inflammatory stimuli such as LPS or heat stroke have been shown to induce NLRP3-dependent inflammasome activation in Kupffer cells, thereby contributing to inflammatory hepatic injury (63–67). Recently, these findings are further supported by studies showing that LPS increased Cx43 expression (68, 69), stimulated ATP release (68), increased NLRP3 inflammasome activation (70), and contributed to septic lethality (68). In light of the notion that diabetes and hyperglycemia are frequently linked to poorer outcomes in experimental (71, 72) and clinical (73–75) sepsis, high glucose levels have also been shown to amplify cytokine-induced Cx43 expression, ATP release, and NLRP3 activation (76).

*In vivo*, endotoxin increases Cx43 expression in the lungs and kidneys (77), but transiently decrease it in the heart (78) (**Table 1**). The mechanisms underlying the tissue-specific regulation of Cx43 expression by endotoxins are not yet understood and remain an intriguing avenue for future investigation. Additionally, Cx43 was found to be upregulated in macrophages cultures by endotoxins and cytokines (56, 60, 62) or septic insults in animals or patients with experimental or clinical peritonitis (68). Conversely, conditional knockout of Cx43 specifically in macrophages reduce LPS-induced ATP release and inflammation in animal models of lethal endotoxemia and CLP sepsis, resulting in improved survival rates (68). Furthermore, genetic deletion or pharmacological inhibition of Cx43 conferred protection against CLP-induced intestinal (79) and lung injury (80), as well as against high-fat diet (HFD)-induced inflammasome activation and inflammation (81), chronic colitis (82), and tubular inflammatory injury (83). Collectively, these findings underscore the compartmentalized regulation of Cx43 during sepsis and suggest that selectively inhibiting Cx43 hemichannels may serve as a potential therapeutic strategy to reduce inflammation in specific tissues.

**Table 1:** Divergent regulation of Cx43 and Panx1 expression in sepsis.

Cells or Tissues	Cx43	Panx1
Macrophages/Monocytes	↑ by LPS, SAA, TNF, and IFN-γ	↑ by LPS, SAA, TNF, and IFN-γ
Lung	↑ by LPS and sepsis	↓ by sepsis
Kidney	↑ by LPS and sepsis	↓ by sepsis
Heart	↓ by LPS and sepsis	↑ by sepsis

downward arrow: decrease; upward arrow: increase.

### Role of Cx43 in the regulation of ATP release

Almost two decades ago, it was proposed that Cx43 hemichannels play a crucial role in facilitating the release of ATP (84), which acts as a pro-inflammatory signal by activating purinergic receptors on adjacent immune cells. In innate immune cells such as macrophages, Cx43 exists predominantly in the form of hemichannels that connect cell interior to extracellular milieu (44, 85, 86). Conditional knockout of Cx43 in lung macrophages led to a significant reduction in ATP release and a diminished calcium response to bleomycin-induced injury (61). Therefore, macrophage Cx43 hemichannel-mediated ATP release is essential for inflammasome activation, and the release of DAMPs (e.g., HMGB1, **Figure 1**) or other inflammasome-dependent cytokines (such as IL-1β and IL-18).

### Divergent effects of Cx43 mimetic peptides on hemichannel activation and sepsis outcomes

**Figure 2:**
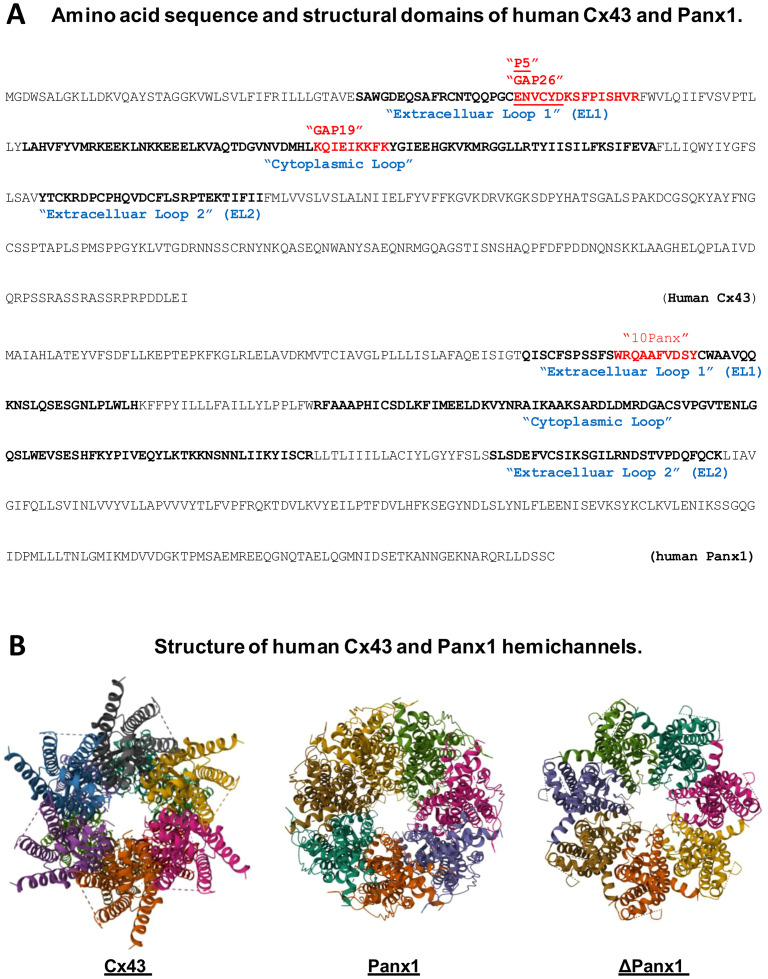
Amino acid sequence and structural domains of human Cx43 and Panx1. **(A)** highlights various mimetic peptides that correspond to specific regions within the extracellular or cytoplasmic loops of Cx43 or Panx1 protein. **(B)** presents the three-dimensional structures of Cx43 and Panx1 hemichannels, including the Panx1 variant lacking the C-terminal tail (ΔPanx1).

Cx43 carries four transmembrane domains that encompass two extracellular loops (ELs), one intracellular cytoplasmic loop, and the intracellular N- and C-termini (**Figure 2A**) (87). The cytoplasmic membrane-bound Cx43 can oligomerize to form hexameric hemichannels (**Figure 2B**) (88), which can dock with hemichannels on adjacent cells to create gap junction (GJ) channels, particularly in non-immune cells of the heart, brain, and vasculature, facilitating intercellular communication (89, 90). Since innate immune cells do not form GJ with one another (86), the upregulation of Cx43 expression may lead to increased hemichannel activity. To explore this, we assessed changes in hemichannel activity using Cx43 mimetic peptides, including GAP26, which corresponds to sequence of the first extracellular loop (EL1) of Cx43 (**Figure 2A**) (91), and GAP19 (**Figure 2A**), derived from a nine-residue segment of the cytoplasmic loop of Cx43 (92). Under calcium-depleted conditions, LPS significantly increased Cx43 hemichannel activity, which was distinctly influenced by pre-incubation with GAP26 and TAT-GAP19 (56). Consequently, these two different Cx43 mimetic peptides, GAP26 and TAT-GAP19, had different effects on LPS-induced hemichannel activation *in vitro*, and varied impacts on the outcomes of lethal sepsis *in vivo* (56).

To further investigate the potential role of Cx43 hemichannels in sepsis, we synthesized a series of hexamer peptides overlapping with the first extracellular loop (EL1) of Cx43 and screened them for their ability to modulate macrophage hemichannel activities. Among these, the cysteine-containing peptide P5 (ENVCYD, **Figure 2A**) significantly inhibited LPS-induced increase in hemichannel activity, as evidenced by a marked reduction in cellular lucifer yellow dye uptake (56). Additionally, the P5 peptide significantly diminished LPS-induced ATP release and trypan blue uptake, indicating its effectiveness in impairing the LPS-mediated elevation of hemichannel activity, cell death and HMGB1 release (56). It is thus possible that crude LPS- and SAA-induced Cx43 upregulation and hemichannel activation may contribute to the efflux of ATP, which activates the purinergic P2X7R to mediate PKR phosphorylation and HMGB1 secretion (**Figure 1**). Furthermore, mimetic peptides such as GAP26 and P5 (ENVCYD) may hold potential as therapeutic agents for the clinical treatment of human sepsis (56).

### Role of Cx43 in innate immune cell communication with non-immune cells

Cx43 hexameric hemichannels can dock with the hemichannels on adjacent cells to form GJ channels to facilitate intercellular communications in various tissues such as the heart, brain, and vasculature (85, 89, 90). Under inflammatory conditions, macrophages form Cx43-containing GJ channel with cardiomyocytes (93), epithelial cells (94), and endothelial cells (62), suggesting an exciting possibility that innate immune cells may communicate with non-immune cells through Cx43-containing GJ channels to orchestrate inflammatory responses. For instance, a subset of alveolar macrophages form Cx43-containing gap junctions with the lung epithelial cells, allowing them to communicate during LPS-induced inflammation (94). This intercommunication resulted in a reduction of neutrophil recruitment and cytokine secretion, thereby serving as a novel immunomodulatory mechanism. Similarly, macrophage and lymphocytes can both express Cx43 and can potentially form GJ channels between innate and adaptive immune cells (95), suggesting a possible role of GJ-mediated communication in immune regulation. Additionally, Cx43 has been implicated in the formation of tunneling nanotubes (96, 97), long membranous channels that can transfer organelles and signaling molecules between immune cells to facilitate intercellular communication over long distances. The Cx43-mediated transfer of cellular contents is especially important during viral infections, as rapid cellular communication is essential for mounting effective inflammatory responses.

## Role of Pannexin 1 as an endogenous regulator of innate immunity in sepsis

Pannexins, originally identified in flatworms as innexin-like membrane channels, also form hexameric hemichannels in vertebrates that similarly enable ATP release (98–100). The Pannexin (Panx) family comprises three members: Panx1, which is widely expressed across diverse tissues; Panx2, primarily restricted to the central nervous system (CNS); and Panx3, predominantly found in skin and skeletal tissues (101).

### Regulation of Panx1 expression and processing

Panx1 is constitutively expressed in quiescent monocytes and macrophages typically at low levels. Exposure to crude LPS or purified SAA markedly elevated Panx1 protein levels in macrophages and monocytes, evidenced by increased Panx1-specific punctate staining in the cytoplasm (57). This finding aligns with prior reports that Panx1 hemichannel expression in microglia was upregulated by proinflammatory cytokines such as TNF, IL-1, and IFN-γ (101). Under non-inflammatory conditions, Panx1 hemichannels remain closed, with the C-terminal tail physically plugging the hemichannel pore from the cytoplasmic side. Upon exposure to inflammatory stimuli, caspase activation can result in the cleavage of the C-terminal tail (102, 103), leading to gradual opening of Panx1 hemichannels and the release of larger molecules like ATP (104, 105). This extracellular ATP acts as an essential danger and “find me” signal (104), attracting innate immune cells to sites of tissue injury or infection, and binding P2X7 receptor to trigger inflammasome activation (51, 103), pyroptosis (106), and the release of HMGB1 (107) and other inflammasome-dependent cytokines such as IL-1β and IL-18 (108–110). Thus, the Panx1-mediated ATP release also plays an important role in promoting innate immune cell chemotaxis (111), inflammasome activation (112), pyroptosis (103, 113), and the inflammatory response (**Figure 1**) (114–116).

Additionally, we observed that both crude LPS and human SAA induced Panx1 release in macrophage cultures (57). This extracellular release of Panx1 was accompanied by the appearance of a lower molecular weight band (12 kDa), detected by a monoclonal antibody specific to the C-terminus of Panx1, suggesting it as a possible degradation product of Panx1. This finding aligns with subsequent report that LPS triggered Casp-11-mediated cleavage of the Panx1 hemichannel in macrophage cultures (103).

While Cx43 and Panx1 share roles in mediating ATP release and promoting inflammation, they display distinct regulatory mechanisms and tissue-specific expression patterns during sepsis. As aforementioned, endotoxin has been shown to reduce Cx43 expression in the heart (78), while increasing it in the kidney and lung (77). In contrast, Panx1 mRNA expression was markedly upregulated in the heart but downregulated in the kidney, lung, and spleen of septic animals (57) (**Table 1**). These findings not only confirm the widespread tissue distribution of Panx1, but also suggest that distinct hemichannel proteins, such as Cx43 and Panx1, are differentially regulated during experimental sepsis. The parallel decrease in circulating Panx1 protein levels and its mRNA expression in macrophage-rich organs, such as the lungs and spleen, suggested that these tissues may contribute to systemic Panx1 accumulation under normal conditions (57).

### Role of Panx1 in the regulation of ATP release

Almost two decades ago, Panx1 was proposed as a signaling molecule essential for Casp-1 processing (117), inflammasome activation, and consequent release of ATP (118) and inflammasome-dependent cytokines (e.g. IL-1β) (51, 109). Subsequently, Panx1 was also identified as a channel for intracellular delivery of muramyl dipeptide, a small molecular component of bacterial peptidoglycan, to trigger Casp-1- and NLRP3-dependent inflammasome activation (119). Beyond inflammasome activation, Panx1 channels also contribute to innate immune cell apoptosis and the release of apoptotic metabolites as signaling molecules (120). For example, Panx1 hemichannels facilitate ATP release from apoptotic cells, guiding macrophages to clear apoptotic bodies (120). This release is triggered by caspase-mediated cleavage of the C-terminal inhibitory domain (102, 120), propelled via an autocrine loop of P2X7 receptor activation (51, 103), and further amplified by Panx1 hemichannel opening (121).

### Role of Panx1 in non-immune cells

Panx1 hemichannels have also been implicated in inflammasome activation in non-immune cells including airway epithelial cells (122), neurons (112), astrocytes (112), and endothelial cells (107). In endothelial cells, Panx1 similarly serves as a channel for ATP release, which activates macrophages through P2X7 receptors, promoting the release of IL-1β and HMGB1 (107). Recently, an Panx1-mediated communication circuit between epithelial cells and macrophages has also been proposed as crucial for promoting epithelial regeneration after injury (123). Suppressing Panx1 expression in epithelial cells with lentivirus-mediated shRNA (122) significantly reduced hypotonic stress-induced ATP release, supporting an important role of Panx1 hemichannels in mediating ATP release in non-immune cells.

### Roles of Panx1 in sepsis and other inflammatory diseases

In animal models of sepsis, Panx1 expression exhibits tissue-specific regulation, with upregulation observed in certain tissues like the heart and downregulation in others, such as the lung and spleen (57) (**Table 1**). This differential regulation of Panx1 expression suggests its tissue-dependent roles in sepsis and other inflammatory diseases. For example, Panx1 has been shown to exacerbate the inflammatory response in animal models of sepsis by promoting the excessive release of ATP, thereby enhancing innate immune cell recruitment and activation. Inhibition of Panx1 conferred protection against sepsis-associated encephalopathy and mortality (113, 124). However, genetic disruption of Panx1 expression rendered animals resistant to lethal endotoxemia (103), but impaired inflammation-mediated microbial clearance (109) and ultimately worsened the outcome of lethal sepsis (125). Similarly, liver-specific knockout of Panx1 also worsened the outcome of lethal endotoxemia (126), suggesting that Panx1 may still be needed for mounting potentially protective innate immune responses against lethal bacterial infections.

The 10Panx, a synthetic peptide that mimics a sequence (WRQAAFVDSY) in the first extracellular loop of Panx1 (**Figure 2A**), is known to inhibit Panx1 hemichannel-mediated ATP release and inflammasome activation in microglia (127) and neurons (51, 128). The administration of 10Panx has been shown to reduce inflammasome activation and attenuate the severity of inflammation in animal models of brain ischemic injury (129), liver toxemia (130, 131), neuropathic pain (132), and epilepsy (133). However, we observed that 10Panx consistently worsened CLP-induced animal lethality, especially when administered at relatively lower doses (10–50 mg/kg) (57). This detrimental effect of 10Panx was associated with an enhancement of the LPS- or SAA-induced hemichannel activation, ATP release and NO production (57). Our finding aligns with the critical role of ATP in PKR-dependent inflammasome activation (42, 59, 103) and NO synthesis (46, 134–136), enforcing the notion that excessive macrophage Panx1 hemichannel activation may contribute to the pathogenesis of lethal systemic inflammation (116). Our finding was also consistent with a previous report that 10Panx did not inhibit macrophage hemichannel activities at a relatively lower concentration (e.g., 100 µM) (137).

### Clinical applicability of Cx43- and Panx1-targeting mimetic peptides

Macrophage hemichannel molecules, such as Cx43 and Panx1, have emerged as pivotal regulators of ATP release, which amplifies innate immune responses in conditions such as sepsis, rheumatoid arthritis, and other inflammatory diseases. Mimetic peptides targeting these hemichannels represent a promising class of therapeutics, offering the potential to selectively modulate macrophage-mediated inflammation. By mimicking endogenous regulatory motifs, these peptides can inhibit pathological hemichannel opening without broadly suppressing innate immune function. This precision allows for effective treatment of diseases characterized by excessive inflammation, such as sepsis and autoimmune disorders, while preserving essential innate immune response required for host defense. Beyond systemic inflammation, hemichannels are also implicated in a range of other conditions, including ischemia-reperfusion injury and neuroinflammation. Mimetic peptides targeting these channels could thus serve as versatile agents for multiple indications, particularly when combining with existing anti-inflammatory agents (e.g., cytokine inhibitors) that could provide synergistic effects. A significant advantage of mimetic peptides lies in their derivation from endogenous proteins, which reduces the risk of immunogenicity and adverse reactions compared to synthetic drugs. This feature enhances their tolerability and facilitates regulatory approval, making them an attractive option for clinical development.

### Challenges in developing mimetic peptides

Despite their promise, the clinical development of mimetic peptides faces several major hurdles. For instance, mimetic peptides often suffer from poor stability *in vivo* due to enzymatic degradation and rapid clearance. Thus, developing stable formulations or introducing structural modifications, such as peptide cyclization or pegylation, is essential to improve their half-life. Additionally, achieving effective delivery to target macrophages in specific tissues remains challenging, particularly for systemic inflammatory diseases where broad biodistribution may dilute their efficacy. While hemichannels are a promising target, Cx43 and Panx1 are ubiquitously expressed in various tissues, including the heart and nervous system. The potentially non-specific inhibition of these channels could result in unintended side effects, such as impaired cardiac conduction or neuronal signaling. Thus, designing peptides with macrophage-specific activity or achieving tissue-specific delivery is essential to avoid these complications. Furthermore, mimetic peptides often exhibit variable pharmacokinetics, complicating dosing strategies. Identifying the appropriate dosing regimen to achieve sustained therapeutic levels without toxicity is a key challenge, especially for conditions like sepsis, where timing is critical. Additionally, obtaining regulatory approval for peptide-based therapies is inherently complex, as these agents often straddle the boundary between small molecules and biologics, requiring comprehensive preclinical testing to demonstrate their safety, efficacy, and manufacturability. Finally, although mimetic peptides are derived from endogenous proteins, modifications to enhance stability or efficacy could inadvertently increase their immunogenic potential, thereby interfering with the efficacy of mimetic peptide therapy.

### Strategies to overcome challenges

To address these challenges, a multidisciplinary approach is needed, such as peptide engineering to incorporate chemical modifications, such as non-natural amino acids or conjugation to delivery vehicles to enhance stability and specificity. Additionally, targeted delivery systems such as nanoparticles, liposomes, or antibody-drug conjugates can be employed to localize peptides to macrophages, thereby minimizing off-target effects and enhancing therapeutic concentration at the disease site. Similarly, combinational therapies of several mimetic peptides as adjuncts to existing treatments (e.g., cytokine inhibitors) can enhance efficacy while mitigating risks. Thus, mimetic peptides targeting macrophage hemichannel molecules hold significant potential as novel therapeutics for inflammatory diseases. Their specificity, versatility, and potential for synergy with existing therapies highlight their clinical applicability. However, challenges related to stability, specificity, and immunogenicity must be addressed to ensure successful translation into clinical practice. Through advancements in peptide engineering, targeted delivery systems, and preclinical validation, these obstacles can be surmounted, paving the way for innovative and effective treatments for inflammatory and immune-mediated diseases.

## Conclusions

Cx43 and Panx1 hemichannels play critical roles in regulating innate immunity during sepsis partly by mediating the release of ATP and other inflammatory signals. Although both hemichannels amplify innate immune responses, their contributions are tissue-specific and disease context-dependent. In septic conditions, Cx43 expression increases in the lung and kidney but decreases in the heart, whereas Panx1 expression is elevated in the heart but reduced in the lung and spleen (**Table 1**). This complex regulation of Cx43 and Panx1 suggests that selective targeting of these hemichannels may offer therapeutic potential for controlling systemic inflammation in tissue-specific fashion. Various inhibitors of Cx43 and Panx1 have shown promise in modulating inflammation across sepsis and other disease models. However, caution is warranted when using mimetic peptides to influence macrophage hemichannel activities, as effects can vary with dose regimens and experimental conditions. Therefore, additional research is necessary to fully elucidate these hemichannels’ multifaceted roles during different phases of sepsis and to develop effective hemichannel-targeted therapies for this devastating condition.
